# Falsely elevated serum testosterone measurements resulting from testosterone topical gel contamination of the venipuncture site: Case Series and retrospective review

**DOI:** 10.1016/j.heliyon.2023.e22819

**Published:** 2023-11-24

**Authors:** Aranza Pinedo Pichilingue, Dina N. Greene, Matthew D. Krasowski

**Affiliations:** a Department of Pathology, University of Iowa Hospitals and Clinics, Iowa City, IA, USA; b Department of Laboratory Medicine, University of Washington, Seattle, WA, USA; c LetsGetChecked Laboratories, Monrovia, CA, USA

**Keywords:** Drug administration routes, Intramuscular injection, Phlebotomy, Pre-analytical phase, Testosterone, Topical administration, Transdermal patch

## Abstract

Testosterone as replacement therapy for male hypogonadism or as gender-affirming hormone therapy for transgender and non-binary patients has increased worldwide. Commonly used formulations of testosterone include intramuscular, transdermal patch, and topical gel, each of which has differing pharmacokinetics and practical challenges. In monitoring testosterone serum concentrations, contamination of the phlebotomy site by testosterone topical gel can lead to supraphysiologic (>1000 ng/dL or 34.7 nmol/L) serum concentrations of testosterone, as demonstrated in a few published case reports. The frequency of this issue is currently not known. The present study involves a retrospective search over a 13-year period across all clinical sites at an academic medical center. Out of 578 unique patients using testosterone topical gel, a total of 48 patients had at least 1 testosterone serum concentration exceed 1000 ng/dL. Documentation in the electronic health record revealed 7 patients where contamination of the phlebotomy site by topical gel was strongly supported as the cause of supraphysiologic testosterone serum concentrations. These included 5 males with primary hypogonadism, 1 male with panhypopituitarism, and a non-binary patient with gender dysphoria. The high testosterone concentrations prompted further work-up, including retesting and endocrinology consultation. There were additional cases of high testosterone serum concentration that may have been falsely elevated due to gel contamination but without sufficient supporting evidence available in the health record. Overall, we present 7 cases of spuriously high testosterone concentrations strongly suspected to be due to venipuncture performed near or at the location of prior testosterone gel application. Gel contamination should be considered as a possible cause of otherwise unexplained high testosterone serum concentration in patients receiving topical testosterone gel formulations. Patient counseling and provider awareness of this potential cause of spuriously high testosterone serum concentrations is important.

## 1 Introduction

Testosterone therapy has increased worldwide in the last decade [[1], [2], [3], [4]]. In the United States, testosterone formulations approved by the Food and Drug Administration (FDA) for testosterone replacement therapy (TRT) include intramuscular injections, transdermal patches, topical gels, implantable subcutaneous pellets, and buccal tablets [[5], [6], [7]]. Oral administration of testosterone is limited by low bioavailability due to poor gastrointestinal absorption and extensive first pass metabolism [[1], [2], [3], [4]]. Intramuscular testosterone cypionate or enanthate have extended duration of action and are relatively inexpensive compared to other testosterone formulations [[5], [6], [7]]. In recent years, topical formulations (gels, transdermal patches) have been increasingly popular, influenced by factors such as patient preference, consumer marketing, and availability of insurance coverage [2,5,[8], [9], [10]].

The use of TRT for treating symptoms of male hypogonadism has been rising in the United States and United Kingdom over the last decade [[1], [2], [3], [4]]. Hypogonadism is estimated to affect one-third of men older than 45 years of age and between 30 and 50 % of men with obesity and/or type 2 diabetes mellitus [[1], [2], [3], [4]]. The diagnosis of hypogonadism is made in men with clinical symptoms and signs of testosterone deficiency (e.g., fatigue, low libido, loss of body hair, loss of muscle mass, erectile dysfunction) plus persistently low serum total testosterone and/or free testosterone concentrations [11,12]. Diagnosis based on a single serum testosterone measurement is discouraged, since about 25 % of men with an initial serum testosterone concentration in the hypogonadal range will exhibit normal testosterone concentrations on subsequent measurements [2,11,12].

Testosterone therapy is also used as gender-affirming hormone therapy (GAHT) in transgender and non-binary patients who desire masculinization, typically in those individuals whose sex assigned at birth is female [13,14]. The main goals of testosterone therapy in this population are to reduce gender dysphoria and promote desired masculinization such as increased muscle mass, deepened voice, and male pattern hair growth. Testosterone as GAHT uses the same testosterone formulations and modalities as for male hypogonadism, although costs and insurance coverage for some formulations are limiting factors for some patients in the United States [15].

Target serum testosterone concentration ranges for TRT or GAHT are a matter of debate, but society guidelines commonly promote the goal of serum testosterone concentrations within the male physiologic range, which is approximately between 400 and 700 ng/dl or 13.9–24.3 nmol/L [[11], [12], [13]]. For hypogonadal men, certain goals such as increased libido may be of highest importance. Regardless of clinical indication, excessively high doses of testosterone have risk of adverse effects, including acne, polycythemia, hypertension, dyslipidemia, and elevated cardiovascular risk [6,7,16,17]. In those with prostate glands, high testosterone concentrations can worsen benign prostatic hyperplasia and theoretically accelerate growth of pre-existing prostate cancer (if present) [17].

Clinical guidelines for management of male hypogonadism and treatment of gender dysphoria often recommend periodic monitoring of serum testosterone (e.g., 1 month after starting therapy, then 3–6 months, and then annually) [11,13]. When monitoring testosterone therapy, it is important to recognize that a variety of factors (e.g., age, genetic polymorphisms, drug-drug interactions, medication adherence or misuse, contamination of sample, fasting status) can influence measured serum testosterone concentration. One of the main physiologic determinants of testosterone secretion is the circadian rhythm, typically resulting in peak concentrations between 7:00 a.m. and 11:00 a.m., followed by a progressive decline to a nadir in the evening, with minimum serum concentrations reached around 8:00 p.m. [18]. In older men, the diurnal pattern is blunted, along with an observed rate of testosterone concentration decline of around 1 %–2 % annually [18]. The influence of circadian rhythms on exogenous testosterone is unknown, but timing of dose and testosterone formulation influence measured testosterone serum concentrations. Therefore, sampling time and age are important factors to consider when monitoring and interpreting serum testosterone in patients on TRT or GAHT. Testosterone concentrations may be suppressed by food intake or glucose. This has led some authorities, including the Endocrine Society in their clinical practice guideline for hypogonadal men, to recommend that the preferred specimen for serum testosterone measurement is a fasting morning specimen [11]. However, there is debate on the requirement for a fasting specimen, as some studies have not shown a significant impact of fasting status on serum testosterone concentrations [19].

Abnormal serum testosterone concentrations should prompt investigation. Testosterone concentrations in patients receiving intramuscular testosterone ester injections are influenced by timing of blood sample relative to last injection, with peak concentrations between 2 and 4 days after injection and a total duration of action of approximately 2 weeks. Testosterone topical gels generally show less variability in serum concentrations compared to transdermal patch [18]. However, artifactually high serum testosterone concentrations in patients receiving topical testosterone gels can occur if the venipuncture site is contaminated by gel during the phlebotomy procedure. This phenomenon was first reported in 2010 as a case report of two instances of spuriously elevated serum testosterone concentrations in a patient resulting from contamination of the antecubital fossa by testosterone gel prior to phlebotomy [20]. A publication in 2013 reported the same phenomenon in two adult patients prescribed testosterone gel for hypogonadism [21]. Testosterone gel contamination has been suspected as a cause for higher testosterone concentrations observed in capillary (fingerstick) relative to venipuncture specimens in some patients [22]. There are also published cases where clinically significant elevations in testosterone concentration resulted from secondary transfer of testosterone gel from one person to another, including children [21,23,24], although controlled studies show this is likely a rare phenomenon [25]. Secondary transfer of testosterone topical gel by laboratory personnel resulting in contamination of reagents or equipment has also been investigated as a potential cause of spurious and unexplained very high testosterone concentrations in a high-volume clinical laboratory [26].

The present retrospective study involving chart review aimed to estimate how often topical testosterone gel contamination could be implicated as a cause for testosterone serum concentrations exceeding 1000 ng/dL without another probable explanation. The search covered a 13-year period (2009–2022) at an academic medical center.

## 2 Materials and methods

### 2.1 Retrospective search strategy

This study was conducted with ethical approval from the University of Iowa Institutional Review Board as a retrospective study with waiver of informed consent. The University of Iowa Hospitals and Clinics (UIHC) are comprised of a 860-bed tertiary/quaternary care medical center with affiliated outpatient clinics at the central campus and at sites throughout the local geographic region. The institutional electronic health record (EHR) is Epic Hyperspace (Epic, Inc.). We utilized EHR reporting tools (Epic Reporting Workbench) [27] to retrieve all serum testosterone measurements from May 1, 2009 to March 31, 2022 along with pharmacy medical records for any testosterone preparations prescribed to patients who had serum testosterone measurements. To try to identify possible cases of testosterone topical gel contamination, we focused chart review on those prescribed topical gel who had at least one serum total testosterone concentration exceeding 1000 ng/dL, the range described in previous case reports of testosterone gel contamination of phlebotomy sites [20,21].

### 2.2 Serum testosterone measurements

During this timeframe, the majority of serum testosterone measurements were performed at the UIHC core clinical laboratory by immunoassay using the Roche Diagnostics Elecsys® Testosterone II assay on either Modular E170 (2009–2012) or cobas e602 analyzers (2013-present). We did not have data on fasting status or the exact timing of serum measurement relative to last dose of testosterone. The analytical measurement range (AMR) and clinical reportable range (CRR) are 12–1500 ng/dL (0.4–52.1 nmol/L). Total imprecision is less than 6.0 %. The performance of the Elecsys Testosterone II assay is comparable to other marketed immunoassays [28,29]. Some serum testosterone measurements (especially those ordered for children or female patients who generally have lower serum testosterone concentrations) were sent to ARUP Laboratories (Salt Lake City, UT) which analyzed by liquid chromatography/tandem mass spectrometry (LC/MS/MS). This assay has an AMR of 1.0–2000 ng/dL (0.03–69.4 nmol/L), CRR up to 10,000 ng/dL (347 nmol/L; with dilution), and total imprecision less than 6.0 % [30]. Some patients had measurements performed by both methodologies at various times.

## 3 Results

### 3.1 Distribution of testosterone concentrations

**Fig. 1:**
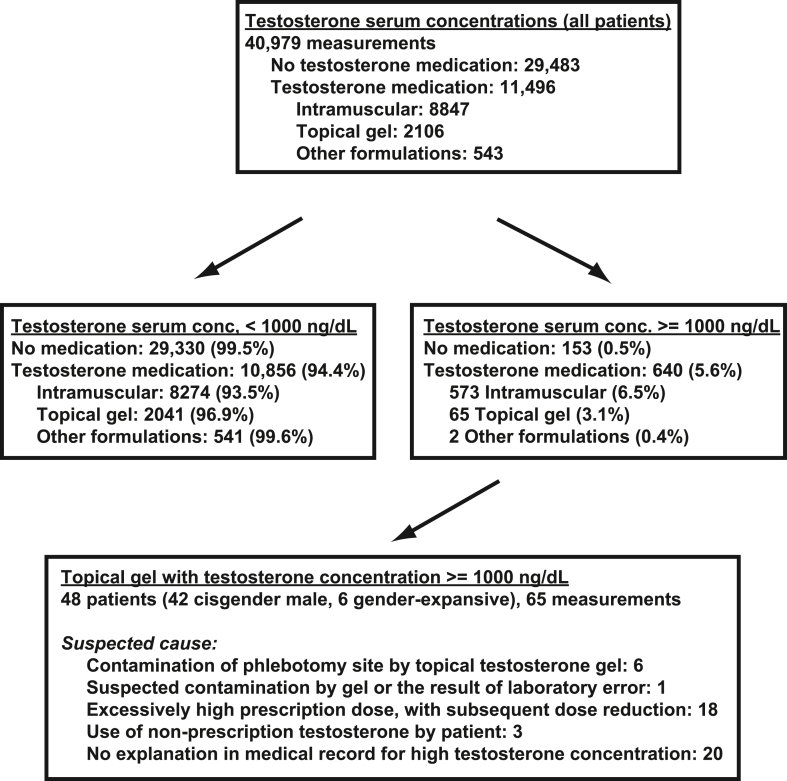
Flow diagram showing number of testosterone serum concentrations for all patients and also broken into categories. The suspected causes in the box on the bottom are derived from extensive chart review of the 48 patients. Abbreviations: conc., concentration.

In the retrospective timeframe, there was a total of 19,623 unique patients with 40,979 total serum testosterone measurements. In terms of analytical methodology, *N* = 35,733 measurements in 16,437 unique patients were performed by immunoassay, while *N* = 5246 measurements in 3817 unique patients were performed by LC/MS/MS method. The majority of testosterone serum measurements were for patients not prescribed any testosterone-containing medications at the time of the blood draw (Table 1; Fig. 1). Intramuscular testosterone was the most common route of administration for those who had testosterone serum concentrations measured (*N* = 8847; 21.6 % of all 40,979 serum testosterone measurements; 77.0 % of the 11,496 measurements for those receiving a testosterone medication at time of measurement), with topical gel being second most common (*N* = 2106; 5.1 % of all testosterone measurements and 18.3 % of measurements for those receiving a testosterone medication at time of measurement). The age distribution was lower in those on intramuscular testosterone (mean age ± SD: 36.6 ± 19.1 years) compared to either topical gel (52.3 ± 15.3 years) or transdermal patch (52.6 ± 15.1 years). Formulations of testosterone other than intramuscular, topical gel, or transdermal patch were uncommon, with only 88 testosterone serum measurements (32 unique patients) in patients prescribed alternate formulations such as mucoadhesive buccal tablets or subcutaneous pellets. Topical gel or transdermal patch formulations were uncommon in patients whose gender identity was recorded as transgender or non-binary in the sexual orientation/gender identity (SOGI) fields in the EHR (Table 1; note that the functionality to supply this information in the institutional EHR only became available in December 2018). Most testosterone serum measurements (39,878 of 40,979, 97.3 %) were performed in patients seen in the outpatient setting.

**Table 1:** Demographics of patients who had testosterone serum concentrations performed.

	Testosterone Formulation^a^				
	No testosterone medication^a^	Intramuscular	Topical gel	Transdermal patch	All other testosterone medications^a^
**Total measurements (unique patients)**	29483 (16691)	8847 (2232)	2106 (578)	455 (105)	88 (32)
**Female**	9023 (6590)	1982 (568)	95 (45)	13 (5)	42 (9)
**Male**	20460 (10101)	6865 (1665)	2011 (533)	442 (100)	46 (22)
**Age (mean ± SD, median)**	37.4 ± 20.5, 34.0	36.6 ± 19.1, 31.7	52.3 ± 15.3, 53.3	52.6 ± 15.1, 55.2	37.9 ± 20.3, 34.7
**Age range**	0.0, >89	0.0, >89	14.3, >89	19.0, 87.1	0.0, >89
**Gender identity (# of measurements)**^b^					
**Transgender female**	1932	29	6	0	4
**Transgender male**	63	2074	30	3	2
**Nonbinary**	199	272	30	3	0
**All others**	27289	6472	2040	449	82
**Location type (# of measurements)**					
**Inpatient**	900	161	30	8	2
**Outpatient**	28583	8686	2076	447	86
**Methodology (# of measurements)**					
**Immunoassay**	24644	8457	2095	450	87
**Mass spectrometry**	4839	390	11	5	1

a “No testosterone medication” indicates that testosterone serum concentration was obtained when patient was not prescribed any medications containing testosterone at time of measurement. “All other testosterone medications” includes testosterone pellets, oral formulation, subcutaneous injection, or unknown (a small number of medication lists for patients documented testosterone but without specifying formulation).

b Gender identity is from the electronic health record field that can be voluntarily supplied by patient. The functionality to do this became available in the medical center in December 2018.

**Fig. 2:**
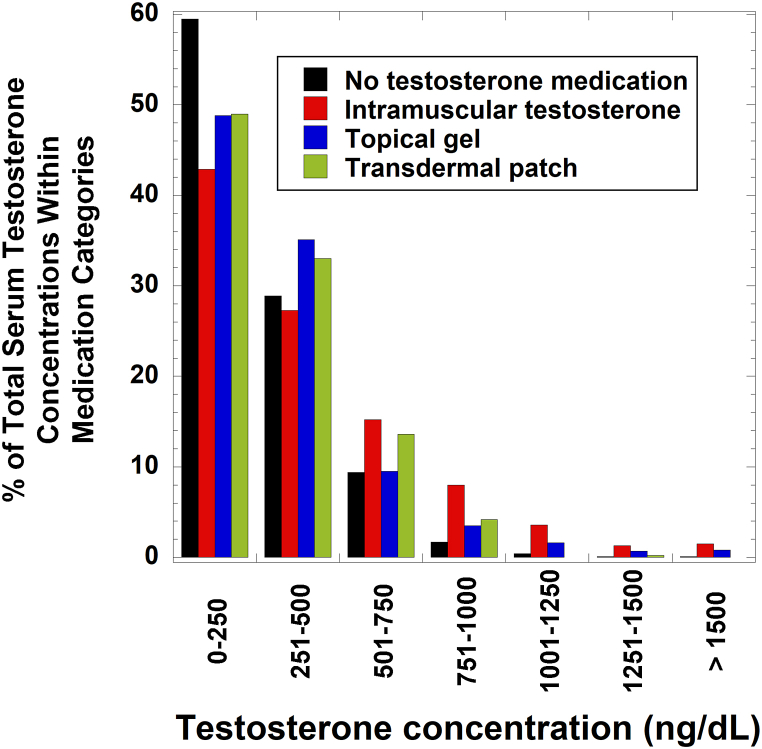
Retrospective distribution of testosterone serum concentrations for patients either prescribed testosterone medications or not on any medications. Data was retrieved from the institutional electronic health record database and compared to pharmacy records. The extracted data included patients not prescribed any testosterone medications at time of serum measurement (*N* = 29483 concentrations, 16691 unique patients) along with those prescribed and actively receiving testosterone by intramuscular formulations (*N* = 8847 concentrations, 2232 unique patients), topical gel (*N* = 2106 concentrations, 578 unique patients), or transdermal patch (*N* = 455 concentrations, 105 unique patients) at the time of serum testosterone measurement. The bars represent percent of the total concentrations in each specific category.

We compared overall distribution of total testosterone serum concentrations among patients who were prescribed testosterone by intramuscular injection, topical gel, or transdermal patch or not prescribed testosterone at all (Fig. 2). Total testosterone serum concentrations 1000 ng/dL or higher were most common in those prescribed testosterone by intramuscular injection (573 of 8847 or 6.5 %) or topical gel (65 of 2106 measurements or 3.1 %), while seen rarely in those using transdermal patch (1 of 455 measurements or 0.2 %) or other routes of administration (1 in 88 or 1.1 %) (intramuscular compared to formulations other than topical gel, *p* < 0.001, Chi square 2 x 2 with Yates's correction for number of tests; topical gel compared to formulations other than intramuscular, *p* < 0.001). Total testosterone serum concentrations of 1500 ng/dL (52.1 nmol/L) or higher were seen in 132 of 8847 measurements (1.5 %) of those prescribed intramuscular injections and 17 of 2106 (0.8 %) of those prescribed topical gel testosterone and were absent with other testosterone formulations. People not documented to be prescribed testosterone-containing medication at the time of serum testosterone measurement had a concentration greater than 1000 ng/dL in 153 of 29483 (0.5 %) measurements and greater than 1500 ng/dL in 27 (0.09 %) measurements.

### 3.2 Patients with elevated testosterone concentrations in conjunction with testosterone topical gel use

We performed chart review of the 65 patients identified in the EHR database as being prescribed topical gel testosterone in conjunction with a testosterone serum concentration of 1000 ng/dL or higher. This chart review narrowed down the list to a group of 48 patients who had clinical notes that verified the patient was actively using testosterone topical gel at the time of the elevated serum testosterone concentration and also provided clinical information around that timeframe as to reason for testosterone therapy (Fig. 1). Of these 48 patients, 42 were cisgender men being treated for hypogonadism (*N* = 42). The remaining 6 patients were transgender men or non-binary individuals receiving gender-affirming hormones. At the time the high serum testosterone concentrations were measured, the average age of patients was 40.3 years with a standard deviation (SD) of ± 17.1 years. The prescriptions for the topical gel in the 48 patients included the following formulations: AndroGel® 1 %, Androgel® 1.62 %, and Testim® 1 %.

Of the 48 individuals on topical gel and with testosterone concentrations of 1000 ng/dL or higher, 7 patients (14.6 %) had documentation in the EHR that mentioned this event and implicated topical gel contamination of the venipuncture site as the likely underlying cause for the elevated testosterone concentration (Fig. 1). We present details of these 7 cases in Section 3.3 below. In the remaining 41 cases (85.4 % testosterone), clinical notes either did not contain sufficient documentation to associate the high testosterone concentrations with topical gel contamination or suspected another cause for the elevated testosterone concentration. From chart review, the other common suspected causes were excessively high prescription testosterone dose (*N* = 18) or use of illicit testosterone supplements by the patient in the addition to the prescription medication (*N* = 3). We were unable to ascertain any suspected cause from the available information in the medical record for 20 of the 48 patients.

### 3.3 Case presentations

#### 3.3.1 Case 1

A 47-year-old male was prescribed 50 mg of testosterone gel to the upper shoulder to treat his symptoms concerning for hypogonadism and was counseled on the risk of secondary transfer of testosterone to others. The patient reported a marked improvement of symptoms after 1 month of treatment, with a total testosterone serum concentration of 498 ng/dL (17.3 nmol/L; reference range: 249–836 ng/dL or 8.6–29.0 nmol/L). At a follow-up appointment 18 months later, his measured total testosterone concentration was 1258 ng/dL (43.7 nmol/L); other laboratory results including hemoglobin/hematocrit were within reference intervals. The patient denied any symptoms or adverse effects attributable to testosterone excess and stated he had not been on any other testosterone medications or over-the-counter supplements of any kind. However, he recalled applying his medication to an area which included the phlebotomy site (left antecubital fossa) shortly before the blood sample was obtained. Thus, the high testosterone value was registered in the patient's records as ‘‘erroneously elevated due to contamination of the sample with testosterone from patient's skin’’.

The patient was instructed to apply his testosterone to the contralateral shoulder to avoid contamination of the needle and to avoid applying his testosterone gel to any areas that are not covered by his T-shirt in the future. Three days later, total testosterone serum concentration was 384 ng/dL (13.3 nmol/L). A second high total testosterone concentration of 1190 ng/dL (41.3 nmol/L) was measured in a follow-up appointment 1 year later. On repeat measurement, the total testosterone was 176 ng/dL (6.1 nmol/L). Documentation in the medical record indicated the second elevated result >1000 ng dL was also likely a consequence of gel contamination at the phlebotomy site. The patient was subsequently lost to follow-up at our institution.

#### 3.3.2 Case 2

A 46-year-old male with a history of hypertension and obesity presented to a family medicine physician for sexual performance difficulties related to erectile dysfunction. In addition to adjusting his antihypertensive medication regimen, the provider ordered a serum total testosterone which was 129 ng/dL (4.5 nmol/L; reference range: 249–836 ng/dL or 8.6–29.0 nmol/L), with a repeat measurement 2 weeks later of 99 ng/dL (3.4 nmol/L). The patient was initially prescribed testosterone transdermal patch but then switched to 50 mg of testosterone gel daily due to lack of insurance coverage for the testosterone patch. On his follow-up appointment, his total testosterone was 1484 ng/dL (51.5 nmol/L). When the physician discussed the wide range of testosterone serum concentrations with the patient, the patient recalled applying a generous amount of testosterone gel on his skin near the phlebotomy site earlier in the morning before the blood draw. A repeated measurement 2 weeks later was 19 ng/dL (0.7 nmol/L); however, the patient admitted being inconsistent with testosterone gel application. His physician emphasized the importance of medication adherence and advised against applying testosterone gel within 8 h before any blood draw. After that appointment, 5 total testosterone concentrations over a 5-year period were all between 279 and 820 ng/dL (9.7–28.5 nmol/L).

#### 3.3.3 Case 3

A 68-year-old male had secondary panhypopituitarism following multiple neurosurgeries to remove a Rathke's cyst. His hormone replacement therapy included 50 mg of testosterone gel three times/week. Over a 7-year period, the patient had 9 total testosterone serum concentrations of 701 ng/dL (24.3 nmol/L; reference range: 193–740 ng/dL or 6.7–25.7 nmol/L) or less, all of which fell within the intended target range for the TRT. He subsequently had a total testosterone serum concentration exceeding 1500 ng/dL (52.1 nmol/L). The primary care physician called the patient to discuss this result, and the patient indicated he had applied testosterone gel earlier in the morning to the same arm in which he received venipuncture for the testosterone concentration. He was advised to obtain testosterone concentrations on days in which he did not apply testosterone gel and to avoid the same arm in which phlebotomy venipuncture was performed. A repeated total testosterone measurement was scheduled a week later, and it also came back elevated with a concentration of 1291 ng/dL (44.8 nmol/L). The patient denied symptoms of testosterone excess, but sample collection for the serum testosterone concentration had again occurred on the same morning as application of testosterone gel to the arm. The patient then did a 3-week trial off of testosterone gel, which resulted in serum total testosterone <5 ng/dL (<0.2 nmol/L). He was restarted on a lower dose of testosterone gel, and the patient transitioned his endocrinology care to a community hospital. Five total serum testosterone concentrations measured at the outside hospital over a 2-year period were all between 138 and 635 ng/dL (4.8–22.0 nmol/L).

#### 3.3.4 Case 4

A 69-year-old male with history of hypertension, type 2 diabetes, chronic kidney disease, and primary hypogonadism was prescribed 25 mg of topical testosterone gel daily for 10 years. A routine serum total testosterone measurement was 1105 ng/dL (38.3 nmol/L; reference range: 193–740 ng/dL or 6.7–25.7 nmol/L), which prompted an endocrinology consult. The patient had been clinically stable, without any symptoms or signs referable to high sex-hormone concentrations. In investigating the case, the endocrinologist found the patient had used his testosterone gel on a large surface area of his bicep the morning his venipuncture was performed. Subsequent follow-up appointments showed total testosterone serum concentrations within the normal range, with values of 411 ng/dL (14.3 nmol/L) and 569 ng/dL (19.7 nmol/L).

#### 3.3.5 Case 5

A 46-year-old male with an 11-year history of primary hypogonadism and erectile dysfunction was prescribed 50 mg of topical testosterone gel/day. His serum testosterone concentrations were between 345 ng/dL (12.0 nmol/L; reference range: 249–836 ng/dL or 8.6–29.0 nmol/L) and 873 ng/dL (30.3 nmol/L) over a 7-year period. A routine serum total testosterone measurement was >1500 ng/dL (52.1 nmol/L). No clinical action was taken on this very high testosterone concentration, and the same testosterone topical gel dose was refilled. The patient subsequently had a second routine serum testosterone concentration of >1500 ng/dL (52.1 nmol/L) 7 months later. Following this measurement, the provider called the patient and learned that the patient had applied the topical gel near the site of phlebotomy approximately 45 min before the blood draw and suspected something similar had happened with the previous elevated measurement. After counseling the patient on how to avoid similar occurrences, subsequent serum testosterone measurements were all less than 700 ng/dL (24.3 nmol/L), and the patient endorsed satisfaction with the clinical effects of the TST.

#### 3.3.6 Case 6

A 49-year-old non-binary patient (assigned female sex at birth) with a history of gender dysphoria started gender-affirming therapy applying 81 mg topical testosterone gel/day. Approximately 3 months later, the patient reported being pleased with the masculinizing effects including growth of facial hair, deepening of the voice, and enlargement of the clitoris. However, a total serum testosterone concentration result exceeded 1500 ng/dL (52.1 nmol/L; reference range if using male values by age: 249–836 ng/dL or 8.6–29.0 nmol/L). Discussion with the patient indicated application of the testosterone gel to the arm shortly before phlebotomy for the testosterone measurement. A follow-up total testosterone concentration was 889 ng/dL (30.8 nmol/L). The patient has since switched to intramuscular testosterone.

#### 3.3.7 Case 7

A 36-year-old male was being followed by medical endocrinology for primary hypogonadism due to a bilateral orchiectomy performed at the age of 3 for cryptorchidism. The patient had erratic adherence to his testosterone therapy of 50 mg topical testosterone gel/day despite symptoms such as low sexual drive and erectile dysfunction. He had total serum testosterone measurements performed every 1–2 years (missing some annual appointments) and had multiple occasions with total serum testosterone concentrations of less than 5 ng/dL (0.2 nmol/L; reference range: 249–836 ng/dL or 8.6–29.0 nmol/L). However, during one encounter he had a total serum testosterone concentration of 1073 ng/dL (37.2 nmol/L). Endocrinology documented that this very high value was likely a result of either gel contamination or a laboratory error. The testosterone dose was reduced to 25 mg daily, but the patient then reported worsening of sexual symptoms despite adhering to this therapy. He had a total serum testosterone concentration measured at another hospital of 7.7 ng/dL (0.3 nmol/L). The topical testosterone gel dosage was increased back to 50 mg daily, resulting in a total serum testosterone concentration of 683 ng/dL (23.7 nmol/L). Three subsequent total serum testosterone concentrations have been between 244 ng/dL (8.5 nmol/L) and 644 ng/dL (22.3 nmol/L).

## 4 Discussion

Measurement of serum testosterone plays an important role in the monitoring of TRT or GAHT [[11], [12], [13]]. Testosterone medication formulation and timing of dosing relative to venous sampling represent important preanalytical factors that can influence serum testosterone concentrations [18]. For instance, serum testosterone concentrations peak 2–4 days after intramuscular injection; sampling serum testosterone within that timeframe will show higher concentrations than at a later time point. There are multiple formulations and doses of injectable testosterone, with testosterone undeconoate having longer action than testosterone enanthate or cypionate [31]. Testosterone formulations also vary across countries. For example, a 1000 mg testosterone undeconoate preparation is marketed in Europe (Nebido®, Bayer, Berkshire, United Kingdom), while the closest equivalent in the United States is a 750 mg testosterone undeconate preparation (Aveed®, Endo International, Malvern, PA, United States) [32]. For those using testosterone topical gel, contamination of the venipuncture site by gel has previously been reported in a few case reports [20,21]. There are also other factors that can impact serum testosterone concentrations, including fasting status [11,33].

Our retrospective review at an academic medical center revealed at least 7 cases over a 13-year period where this phenomenon was suspected to have resulted in a serum testosterone concentration of 1000 ng/dL or higher. In 6 of our cases (patients # 1–6), the likelihood of contamination of blood samples with testosterone gel was identified (although not always promptly) and documented by physicians. For patient #7, a change to a lower medication dose was made before later considering contamination as the source of high serum testosterone concentration. This change in dose led to inadequate therapy, with a later increase in dose by another healthcare provider. We think it is likely that testosterone gel contamination was the underlying cause for at least some of the 41 remaining subjects who had one or more serum testosterone concentrations of 1000 ng/dL or higher. However, documentation in the medical record was not sufficient to provide certainty.

In a multicenter, randomized, double-blind, placebo-controlled study, the serum testosterone concentrations observed in hypogonadal men who received titrated doses of Androgel® 1.62 % (the gel with the highest testosterone concentration available) oscillated between 300 ng/dL (10.4 nmol/L) and 1000 ng/dL during 182 days of daily testosterone therapy to the upper arms/shoulders or abdomen [34]. No concentrations above 1000 ng/dL were observed in the participants. Thus, this suggests that patients receiving testosterone therapy with testosterone gels (of 1.62 % concentration or less) in accordance with recommendations stipulated in current clinical guidelines are expected to exhibit serum testosterone concentrations within the eugonadal range or supraphysiologic concentrations below 1000 ng/dL.

Moreover, a retrospective study over an 18 year-period showed significant differences in serum testosterone concentrations achieved by therapy between the two most commonly prescribed formulations [35]. Their study showed that 62 transgender men receiving GAHT with transdermal testosterone (patch or topical gel) had significantly lower median concentrations of serum total testosterone (326 ng/dL) when compared with those receiving injectable preparations (525 ng/dL). Additionally, patients receiving testosterone by injection attained desired physical changes faster than patients in testosterone therapy with topical gel formulations.

Another study demonstrated that patients prescribed injectable testosterone therapy had higher serum testosterone concentrations at the beginning of treatment compared to patients prescribed topical gel, with stable serum testosterone concentrations over time [31]. On the other hand, patients prescribed topical testosterone therapy achieved lower serum testosterone concentrations at the beginning of treatment, but significant serum testosterone fluctuations were more likely to be observed. Comparative studies involving newer testosterone formulations such as pellets or mucosal adhesive buccal tablets are limited [18].

It is worth noting that product insert material for testosterone gels may not mention the risk of contamination to phlebotomy. For example, package inserts for three testosterone gel products marketed in the United States or Europe (Testim®, Paladin Labs, Inc, Quebec, Canada; AndroGel® 1 % or 1.62 %, ASCEND Therapeutics, United States; Tostran® 2 %, PHARBIL Waltrop GmbH, Germany) all have specific discussion of the risk of secondary transfer of testosterone gel to others (including women and children) but do not mention risk of contaminating phlebotomy venipunture sites [[36], [37], [38]]. Recommended sites of application of these products described in the package inserts also vary: skin of shoulders and/or upper arms (Testim®) [37]; shoulders and upper arms that will be covered by a short-sleeve t-shirt (AndroGel® 1.62 %) [38]; shoulders, upper arms, or stomach area (abdomen) that will be covered by a short-sleeve t-shirt (AndroGel® 1 %) [38]; or rubbed into abdomen or each thigh, ideally alternating days between abdomen and thighs (Tostran® 2 %) [36]. Location of application will influence risk of contaminating venipuncture sites [[20], [21], [22]].

Patients should be encouraged to adhere to their prescribed regimen of TST/GAHT. For those prescribed topical gel, they should always be counseled about the proper anatomic site to apply the testosterone gel (in the shoulder and upper portion of the arm, carefully avoiding the phlebotomy site). The risk of secondary exposure can be limited by hand-washing immediately after application of the gel, covering of the area with clothing, and limiting direct contact of skin with family members. Ideally, serum testosterone concentrations should be measured at least 8 h since last application of the gel.

The present study shows that supraphysiological testosterone serum concentrations from topical gel testosterone contamination is a factor that can affect multiple patients, particularly at centers that frequently prescribe testosterone topical gel. Future studies can aim to replicate this finding with controlled application of various formulations of testosterone topical gel to sites used for phlebotomy. Health care providers should be aware of the possibility of topical gel contamination to avoid unnecessary downstream consequences of apparent supraphysiologic serum testosterone concentrations. Limitations of the present study include retrospective study at a single medical center and sparse medical record documentation in many of the cases. The present study does not have data on fasting status of patients who had serum testosterone measurements performed or on the exact timing of last testosterone dose to time of venipuncture. There is also the challenge that some patients sought care at clinical sites other than UIHC where medical information may not have been transferred to UIHC medical records. Lastly, the current study was performed in the United States, and there are differences in the formulation and available doses of marketed testosterone prescriptions in other countries.

## 5 Conclusions

Overall, we present 7 cases where contamination of the phlebotomy site by testosterone gel was strongly suspected to have caused spuriously high serum testosterone concentrations. For those prescribing testosterone gels, patient counseling and provider awareness of this potential cause of spuriously high testosterone serum concentrations is important.

## Funding statement

There was no external funding for this study.

## Ethics approval

This study was conducted with ethical approval from the University of Iowa Institutional Review Board as a retrospective study with waiver of informed consent with the approval number 202203611.

## Data availability statement

Data associated with this study has been deposited in Mendeley Data, Pinedo Pichilingue, Aranza; Greene, Dina; Krasowski, Matthew (2023), “Serum testosterone concentrations and testosterone medications”, Mendeley Data, V1, doi: 10.17632/nvc843m5tx.1, https://data.mendeley.com/datasets/nvc843m5tx/1.

## CRediT authorship contribution statement

**Aranza Pinedo Pichilingue:** Writing – review & editing, Writing – original draft, Investigation, Formal analysis, Conceptualization. **Dina N. Greene:** Writing – review & editing, Investigation, Formal analysis. **Matthew D. Krasowski:** Writing – review & editing, Writing – original draft, Supervision, Methodology, Conceptualization.

## Declaration of competing interest

The authors declare that they have no known competing financial interests or personal relationships that could have appeared to influence the work reported in this paper.
