# Supplementary material for: Falsely elevated serum testosterone measurements resulting from testosterone topical gel contamination of the venipuncture site: Case Series and retrospective review
Source: Heliyon. 2023 Nov 24;9(12):e22819. doi: 10.1016/j.heliyon.2023.e22819 (PMC10716548; doi:10.1016/j.heliyon.2023.e22819)
Supplement: Multimedia component 1 [file mmc1.pdf]

IRB#: 202203611
Study Title: Causes of Very Elevated Testosterone Values
PI Name: Matthew Krasowski

Below is approval information for any Modifications, Continuing Reviews, or New Project Forms. This includes approval codes, approval memos and other review information if applicable. The most recent forms are listed first.

Mod approved on 07/12/22 (Electronically signed by IRB Chair: Brian Bishop, CIP, MA on 07/12/22 0953)

Administrative Codes

- MODIFICATION Expedited per 45 CFR 46.110(b)(2)

Renewal Requirement Type

Biennial Renewal

Approval Memos

Attachment Name	Category	Ver	Size			Attached
approval-memo.rtf (/hawkirb/attachment /view.page?action=download&oid=87631080)	Approval Form	1	990 k	E	">	07/12/22

Other Reviews

No other reviews were needed for this form.

New approved on 04/01/22 (Electronically signed by IRB Chair: Catherine Woodman, MD on 04/01/22 1258)

Administrative Codes

- EXPEDITED per 45 CFR 46.110(b)(1), Category 5
- WAIVER of Documentation of consent per 45 CFR 46.117(c)(2)
- WAIVER of Elements of Consent per 45 CFR 46.116 (f) General waiver or alteration of consent
- HIPAA: Full waiver of HIPAA Authorization per 45 CFR 46.164.512(i)(2)

Renewal Requirement Type

Biennial Renewal

Approval Memos

Attachment Name	Category	Ver	Size			Attached
full-hipaa-waiver.rtf (/hawkirb/attachment /view.page?action=download&oid=85795581)	Approval Form	1	1 M	E	">	04/01/22
approval-memo.rtf (/hawkirb/attachment /view.page?action=download&oid=85795579)	Approval Form	1	990 k	E	">	04/01/22

Other Reviews

No other reviews were needed for this form.

THE UNIVERSITY OF IOWA

Human Subjects Office/
Institutional Review Board (IRB)
105 Hardin Library for the Health Sciences
600 Newton Road
Iowa City, Iowa 52242-1098
319-335-6564 *Fax* 319-335-7310
irb@uiowa.edu
http://research.uiowa.edu/hso

IRB ID #: 202203611

To: Matthew Krasowski

From: IRB-01 DHHS Registration # IRB00000099,
Univ of Iowa, DHHS Federalwide Assurance # FWA00003007

Re: Causes of Very Elevated Testosterone Values

Protocol Number:
Protocol Version:
Protocol Date:
Amendment Number/Date(s):

Approval Date: 04/01/22

Next IRB Approval Due Before: 03/31/24

Type of Application:

- ☒ New Project
- ☐ Continuing Review
- ☐ Biennial Review
- ☐ Modification

Type of Application Review:

- ☐ Full Board:
  Meeting Date:
- ☒ Expedited
- ☐ Exempt

Approved for Populations:

- ☐ Children
- ☐ Prisoners
- ☐ Pregnant Women, Fetuses, Neonates

Source of Support:

Investigational New Drug/Biologic Name:
Investigational New Drug/Biologic Number:
Name of Sponsor who holds IND:

Investigational Device Name:
Investigational Device Number:
Sponsor who holds IDE:

The following documents have been submitted for the above review and approval:

Assurance Document	IRB_assurance_testosterone_033122.pdf

This approval has been electronically signed by IRB Chair:

OFFICE OF THE VICE PRESIDENT FOR RESEARCH

Catherine Woodman, MD
04/01/22 1258

As Principal Investigator, you are responsible for ensuring this project is conducted in compliance with all applicable federal, state, and local laws and regulations, institutional policies, and requirements of the IRB, which include, but are not limited to, the following:

IRB Approval: IRB approval indicates that this project meets the regulatory requirements for the protection of human subjects. The research is approved to be conducted as described in the HawkIRB application. The addition or omission of study activities is not permitted without prior IRB review and approval. IRB approval does not absolve the principal investigator from complying with other institutional, collegiate, or departmental policies or procedures.

Agency Notification: If this is a New Project or Continuing Review application and the project is funded by an external government or non-profit agency, the original HHS 310 form, “Protection of Human Subjects Assurance Identification/IRB Certification/Declaration of Exemption,” has been forwarded to the UI Division of Sponsored Programs, 100 Gilmore Hall, for appropriate action. You will receive a signed copy from Sponsored Programs.

Recruitment: Your IRB application has been approved for recruitment of subjects not to exceed the number indicated on your application form. The IRB has approved all recruitment strategies described in the application. It is not necessary to use all of thesestrategies, but no additional recruitment strategies may be used without IRB approval.

Biennial Review: Eligible, non-exempt, studies that do not require an annual continuing review will require a Biennial check in until the project is closed in HawkIRB by the Principal Investigator. The required Biennial check in will include a very brief, seven question check every other year. You are responsible for submitting a Biennial Review in sufficient time for review.

Modifications: Any change in this research project or materials must be submitted on a Modification application to the IRB for prior review and approval, except when a change is necessary to eliminate apparent immediate hazards to subjects. The investigator is required to promptly notify the IRB of any changes made without IRB approval to eliminate apparent immediate hazards tosubjects using the Modification/Update Form. Modifications requiring the prior review and approval of the IRB include but are not limited to: changing the protocol or study procedures, changing investigators or funding sources, changing the Informed Consent Document, increasing the anticipated total number of subjects from what was originally approved, or adding any new materials (e.g., letters to subjects, ads, questionnaires).

Unanticipated Problems Involving Risks: You must promptly report to the IRB any serious and/or unexpected adverse experience, as defined in the UI Investigator’s Guide, and any other unanticipated problems involving risks to subjects or others. The Reportable Events Form (REF) should be used for reporting to the IRB. Reports from the investigator to the IRB must be submitted via HawkIRB within ten working days of the event or within 10 working days of the PI becoming aware of the event.

Audits/Record-Keeping: Your research records may be audited at any time during or after the implementation of your project. Federal and University policies require that all research records be maintained fora period of three (3) years following the close of the research project. For research that involves drugs or devices seeking FDA approval, the research records must be kept for a period of three years after the FDA has taken final action on the marketing application. For research that involves Protected Health Information(PHI) under HIPAA, the research records must be kept for a period of six (6) years folling the close of the research project.

Additional Information: Complete information regarding research involving human subjects at The University of Iowa is available in the “Investigator’s Guide to Human Subjects Research.” Research

investigators are expected to comply with these policies and procedures, and to be familiar with the University's Federalwide Assurance, the Belmont Report, 45CFR46, and other applicable regulations prior to conducting the research. These documents and IRB application and related forms are available on the Human Subjects Office website or are available by calling 335-6564.

THE UNIVERSITY OF IOWA

Human Subjects Office/
Institutional Review Board (IRB)
105 Hardin Library for the Health Sciences
600 Newton Road
Iowa City, Iowa 52242-1098
319-335-6564 *Fax* 319-335-7310
irb@uiowa.edu
http://research.uiowa.edu/hso

IRB ID #: 202203611

To: Matthew Krasowski

From: IRB-01 DHHS Registration # IRB00000099,
Univ of Iowa DHHS Federalwide Assurance # FWA00003007

Re: Causes of Very Elevated Testosterone Values

Protocol Number:
Protocol Version:
Protocol Date:
Amendment Number/Date(s):

Approval Date: 04/01/22 (Expedited)

This project has been granted a full waiver of HIPAA Authorization based on the documentation provided by the researcher in the HawkIRB application Section IV and the assurance document signed by the Principal Investigator.

This full waiver of authorization satisfies the following criteria:
(1) The use or disclosure of the requested information involves no more than a minimal risk to the privacy of individuals based on, at least, the presence of the following elements:
(a) An adequate plan to protect the identifiers from improper use and disclosure
(b) An adequate plan to destroy the identifiers at the earliest opportunity consistent with conduct of the research, unless there is a health or research justification for retaining the identifiers or such retention is otherwise required by law; and
(c) Adequate written assurances that the requested information will not be reused or disclosed to any other person or entity, except as required by law, for authorized oversight of the research study, or for other research for which the use or disclosure of the requested information would be permitted by the Privacy Rule;
(2) The research could not practicably be conducted without the waiver or alteration; and
(3) The research could not practicably be conducted without access to and use of the requested information.

This approval has been electronically signed by IRB Chair:
Catherine Woodman, MD
04/01/22 1258
